# Effects of sacubitril/valsartan on the functional capacity of real-world patients in Italy: the REAL.IT study on heart failure with reduced ejection fraction

**DOI:** 10.3389/fcvm.2024.1347908

**Published:** 2024-05-10

**Authors:** Filippo Maria Sarullo, Cinzia Nugara, Silvia Sarullo, Massimo Iacoviello, Gabriele Di Gesaro, Daniela Miani, Mauro Driussi, Michele Correale, Claudio Bilato, Andrea Passantino, Erberto Carluccio, Alessandra Villani, Luca Degli Esposti, Chiara D’Agostino, Elena Peruzzi, Simone Poli, Andrea Di Lenarda

**Affiliations:** ^1^ U.O.S.D. di Riabilitazione Cardiovascolare Ospedale Buccheri La Ferla Fatebenefratelli, Palermo, Italy; ^2^ School of Sport Medicine and Physical Exercise Medicine, Department of Biomedicine, Neurosciences and Advances Diagnostic, University of Palermo, Palermo, Italy; ^3^ Department of Surgical and Medical Sciences, University of Foggia, Foggia, Italy; ^4^ U.O. Cardiologia IRCCS ISMETT Palermo, Palermo, Italy; ^5^ SOC Cardiologia, Dipartimento Cardiotoracico, Azienda Sanitaria Universitaria Friuli Centrale Ospedale S. Maria della Misericordia, Udine, Italy; ^6^ SC Universitaria di Cardiologia AOU “Ospedali Riuniti”, Foggia, Italy; ^7^ U.O.C. Cardiologia Azienda ULSS 8 Berica—Ospedali dell’Ovest Vicentino, Arzignano, Italy; ^8^ Istituti Clinici Scientifici Maugeri IRCCS, Cardiac Rehabilitation Unit of Bari Institute, Bari, Italy; ^9^ Cardiologia e Fisiopatologia Cardiovascolare, Azienda Ospedaliera Universitaria “Santa Maria della Misericordia”, Perugia, Italy; ^10^ UO Cardiologia, Dipartimento di Scienze Cardiovascolari, Neurologiche, Metaboliche, Istituto Auxologico Italiano IRCCS, Milano, Italy; ^11^ CliCon S.r.l., Bologna, Italy; ^12^ Novartis Farma SpA, Milan, Italy; ^13^ Cardiovascular Center, University Hospital and Health Services of Trieste, Trieste, Italy

**Keywords:** functional capacity, cardiopulmonary exercise testing, heart failure with reduced ejection fraction, real-world practice, sacubitril/valsartan

## Abstract

**Background:**

Heart failure (HF) significantly affects the morbidity, mortality, and quality of life of patients. New therapeutic strategies aim to improve the functional capacity and quality of life of patients while controlling HF-related risks. Real-world data on both the functional and cardiopulmonary exercise capacities of patients with HF with reduced ejection fraction upon sacubitril/valsartan use are lacking.

**Methods:**

A multicenter, retrospective, cohort study, called REAL.IT, was performed based on the data collected from the electronic medical records of nine specialized HF centers in Italy. Cardiopulmonary exercise testing was performed at baseline and after 12 months of sacubitril/valsartan therapy, monitoring carbon dioxide production (VCO_2_) and oxygen consumption (VO_2_).

**Results:**

The functional capacities of 170 patients were evaluated. The most common comorbidities were hypertension and diabetes (i.e., 53.5 and 32.4%, respectively). At follow-up, both the VO_2_ peak (from 15.1 ± 3.7 ml/kg/min at baseline to 17.6 ± 4.7 ml/kg/min at follow-up, *p* < 0.0001) and the predicted % VO_2_ peak (from 55.5 ± 14.1 to 65.5 ± 16.9, *p* < 0.0001) significantly increased from baseline. The VO_2_ at the anaerobic threshold (AT-VO_2_) increased from 11.5 ± 2.6 to 12.5 ± 3.3 ml/kg/min (*p* = 0.021), and the rate ratio between the oxygen uptake and the change in work (ΔVO_2_/Δwork slope) improved from 9.1 ± 1.5 to 9.9 ± 1.6 ml/min/W (*p* < 0.0001).

**Conclusions:**

Sacubitril/valsartan improves the cardiopulmonary capacity of patients with HFrEF in daily clinical practice in Italy.

## 1 Introduction

Heart failure (HF) affects approximately 64 million people worldwide (1) and is recognized as a global pandemic. HF imposes a significant burden on the morbidity and mortality of patients, showing reductions in their functional capacities and quality of life and requiring high societal and healthcare costs (2). The HF prevalence is reported to be approximately 1.7% in Italy and increases sharply with age (3, 4). Reducing the social and economic burdens of HF has become a major public health concern worldwide. Ongoing studies prioritize innovative therapeutic approaches to enhance the clinical condition, functional capacity, and quality of life of patients while minimizing the likelihood of HF-related hospitalization and mortality (5).

However, the underuse and underdosing of guideline-directed medical therapy in daily clinical practice continue to hinder the achievement of optimized HF management (6). Sacubitril/valsartan therapy simultaneously inhibits neprilysin and blocks the AT1 receptor via sacubitril and valsartan, respectively, and has been shown to provide substantial clinically relevant cardiovascular (CV) benefits. It also improves the symptoms and physical constraints of HF when compared to the standard treatment that uses enalapril in patients experiencing symptomatic HF with reduced ejection fraction (HFrEF) (7). The improvement in the exercise tolerance and the VO_2_ peak observed during sacubitril/valsartan therapy is likely attributed to the combined effects of the AT1 receptor antagonist and the neprilysin inhibitor. Through the action on AT1, valsartan causes vasodilation and volume reduction, while the sacubitril inhibition of neprilysin reduces the breakdown of vasoactive peptides, including natriuretic peptides, bradykinin, and adrenomedullin. The increased levels of these substances promote vasodilation and natriuresis, amplifying the system of vasoactive substances, such as bradykinin, adrenomedullin, endothelin-1, substance P, and angiotensin II. This amplification of the vasodilatory and natriuretic effects likely contributes to the observed improvement in exercise tolerance during sacubitril/valsartan therapy (8).

The most recent guidelines of the European Society of Cardiology (ESC) for the treatment of HFrEF include sacubitril/valsartan in the recommended sequence of different classes of medical therapies for HF (5).

Although sacubitril/valsartan is an established mainstay of HFrEF therapy and supported by strong evidence-based clinical trial data, evidence for its use and the characteristics and resource utilization of patients treated in real-world settings of routine practice are more limited, as are the effects of sacubitril/valsartan on treatment outcomes. Patients in real-world clinical settings are likely to be older and frailer than those included in randomized clinical trials because the latter usually present more stringent inclusion and exclusion criteria. Therefore, real-world data are needed to provide valuable support for the efficacy and safety findings of Phase 3 clinical studies; however, data regarding the use of sacubitril/valsartan in clinical routine in Italy are currently limited (9).

In light of this, a multicenter, retrospective, cohort study, called the REAL.IT, was performed by utilizing data gathered from Italian outpatient specialist clinics to define the clinical characteristics of and outcomes for Italian patients with HFrEF upon sacubitril/valsartan therapy (10, 11). The exploratory objective of REAL.IT, which was the focus of this study, was to describe the outcome of using sacubitril/valsartan on the functional capacity and cardiopulmonary exercise capacity of the Italian real-world cohort of patients with HFrEF.

## 2 Methods

### 2.1 Study design and participants

For the retrospective REAL.IT study, the patients' data were retrieved from the electronic medical records (EMR) and administrative databases of nine hospitals in Italy that specialize in the management of HF. Appendix 1 lists the principal investigators involved in the study with the centers they belong to.

The study participants were adults aged ≥18 who were diagnosed with HF and were attending the outpatient clinic at any of the nine centers involved in this work. The patients had to be prescribed sacubitril/valsartan at least once in the time window of 1 October 2016 (time of launch in Italy)–30 June 2019 (inclusion period). The rationale for the 30 June 2019 cut-off date was to allow for a follow-up period of ≥1 year at the time of data extraction on 30 June 2020.

The index date and the characterization period were defined as the date of the first prescription of sacubitril/valsartan during the inclusion period and the 6 months before the index date, respectively. The baseline characteristics of the patient population were evaluated during the characterization period. A comparison between the characterization and follow-up periods was conducted only for patients with available information on the variables of interest in both periods. If there was missing information regarding age or sex, the patients were excluded from the analysis.

The study adhered to the principles of the Declaration of Helsinki and was submitted for review to the ethics committee of each participating center in accordance with the Italian regulations governing observational studies.

### 2.2 Data collection and cardiopulmonary evaluation

As previously reported, data were collected not earlier than 6 months before the index date. The demographic features, previous clinical history, functional parameters, comorbidities, and pharmacological treatments for HF of the general study population have already been reported (10). Given the use of secondary data, safety monitoring was not performed.

Cardiopulmonary exercise testing (CPET) was performed at baseline before the initiation of the therapy and after 12 months. All CPET sessions were conducted following a previously reported protocol (8) using the Vmax 2900 device (SensorMedics, Yorba Linda, CA, USA). All throughout the examination, the test continuously monitored every individual's 12-lead electrocardiogram (ECG) and oxygen saturation levels with a pulse oximeter. The patients were advised to persist with physical activity until they experienced muscle exhaustion and/or shortness of breath. The output parameters included the anaerobic threshold (AT) determined through the V-slope analysis, the increased VO_2_ per watt of work (ΔVO_2_/Δwork), the slope of the relationship between minute ventilation and CO_2_ production (VE/VCO_2_ slope), and the ratio between the dead space volume and the tidal volume (RV/VT) calculated using Jones' prediction equation (12), VO_2_, ventilation, tidal volume, and RV/VT ratio at peak exercise (8).

### 2.3 Statistical analysis

The data were analyzed as already reported (10, 11). Briefly, *χ*^2^ and paired *t*-tests were used to compare categorical and continuous variables, respectively. A *p*-value of <0.05 was considered statistically significant. STATA SE version 12.0 (StataCorp LLC, College Station, TX, USA) was used for the statistical analyses. Microsoft SQL Server 2012 was used for data management.

## 3 Results

The overall patient population of REAL.IT consisted of 948 adult HF patients. The baseline demographic, clinical characteristics, and clinical outcomes of the overall population have already been reported (10).

The functional capacities of 170 patients were evaluated. Table 1 reports their demographic and clinical features. In summary, 85.9% of the patients were males with a mean [standard deviation (SD)] age of 60.1 (10.2) years. The mean LV ejection fraction (LVEF) was 28 ± 5.7%. The major comorbidities reported were hypertension (53.5%) and diabetes (32.4%). Of the 126 patients with available data, 94 (74.6%) had an implantable device, and 30/48 patients (62.5%) had ischemic heart disease. As for drug therapy, 83% of the patients were taking furosemide; 70%, ACEIs; 30%, ARBs; 83%, mineralocorticoid antagonists; 94%, β-blockers; 18%, ivabradine; and 5%, digoxin. The baseline characteristics of the subgroup considered for this analysis were similar to those of the whole sample described in the related publication (10).

**Table 1:** Baseline demographic and clinical characteristics of patients included in the functional capacity analyses (*N* = 170)^a^.

Characteristic	
Age, years, mean ± SD	60.1 (10.2)
Gender, *n* (%)	
Male	146/170 (85.9)
Female	24/170 (14.1)
Ischemic heart disease, *n* (%)	30/48 (62.5)
PCI/CABG, *n* (%)	27/48 (56.3)
Moderate or severe mitral or aortic valvulopathy, *n* (%)	7/48 (14.6)
Implanted prosthetic valve, *n* (%)	6/44 (13.6)
Devices (ICD/CRT), *n* (%)	94/126 (74.6)
Atrial fibrillation, *n* (%)	31/170 (18.2)
Diabetes mellitus, *n* (%)	55/170 (32.4)
Hypertension, *n* (%)	91/170 (53.5)
CKD in patients with available data during the characterization period, *n* (%)	13/46 (28.3)
CKD (considering patients with available data for diagnosis of CDK and those for whom CDK was deduced from the creatinine value considering the eGFR), *n* (%)	84/161 (52.2)
Duration of HF disease, years^b^, mean ± SD	8.0 ± 7.1

CABG, coronary artery bypass grafting; CKD, chronic kidney disease; CRT, cardiac resynchronization therapy; eGFR, estimated glomerular filtration rate; HF, heart failure; ICD, implantable cardioverter defibrillator; PCI, percutaneous coronary intervention; and SD, standard deviation.

^a^ Patients with available data during the characterization period, which was 6 months before the index date, the date of the first prescription of sacubitril/valsartan during the inclusion period. Patient follow-up was done for at least 12 months.

^b^ *N* = 152 patients with available data.

When the baseline data were compared to those from a 12-month follow-up, treatment with sacubitril/valsartan improved the cardiopulmonary functional capacity of patients (Table 2). There were highly significant improvements in the peak and predicted peak oxygen consumption (VO_2_ peak) and the VE/VCO_2_ slope between the baseline and the 12-month follow-up. The mean peak VO_2_ improved from 15.1 ± 3.7 ml/kg/min at the baseline to 17.6 ± 4.7 ml/kg/min at the follow-up (*p* < 0.0001). The mean predicted % peak VO_2_ improved from 55.5 ± 14.1 to 65.5 ± 16.9 (*p* < 0.0001). Lastly, the mean minute ventilation per unit carbon dioxide production (VE/VCO_2_) slope decreased from 33.2 ± 6.1 to 30.7 ± 6.1 (*p* = 0.0009). Correspondingly, there were improvements observed in the VE/VCO_2_ slope > 34 (*p* = 0.015); the AT at the oxygen consumption peak (AT-VO_2_) increased from 11.5 ± 2.6 to 12.5 ± 3.3 ml/kg/min (*p* = 0.021); and the changes in the oxygen uptake and work rate ratio (ΔVO_2_/Δwork) improved from 9.1 ± 1.5 to 9.9 ± 1.6 ml/min/W (*p* < 0.0001). There were also corresponding improvements in the oxygen pulse, peak ventilation, tidal peak volume, and ventilatory oscillation (Table 2).

**Table 2:** Cardiopulmonary exercise stress testing (CPET) data at the baseline and after a 12-month follow-up (*N* = 170)^a^.

CPET data	Baseline	Follow-up	*p*-value
VO_2_ peak (ml/kg/min), mean ± SD	15.1 ± 3.7	17.6 ± 4.7	<0.0001
VO_2_ peak (predicted %), mean ± SD	55.5 ± 14.1	65.5 ± 16.9	<0.0001
V_E_/VCO_2_ slope, mean ± SD	33.2 ± 6.1	30.7 ± 6.1	0.0009
V_E_/VCO_2_ slope >34 (*n*, %)	58 (43)	38 (28)	0.015
RQ, mean ± SD	1.13 ± 0.11	1.14 ± 0.10	0.45
Watt (peak), mean ± SD	74 ± 25	90 ± 32	<0.0001
AT-VO_2_ (ml/kg/min), mean ± SD	11.5 ± 2.6	12.5 ± 3.3	0.021
AT-VO_2_ predicted (ml/kg/min), mean ± SD	42.8 ± 12	47 ± 13.4	0.020
AT not achieved (*n*, %)	33 (25)	24 (18)	0.23
O_2_ pulse (ml/beat), mean ± SD	11.7 ± 3.1	13.4 ± 3.8	0.0002
ΔVO_2_/Δwork (ml/min/W), mean ± SD	9.1 ± 1.5	9.9 ± 1.6	<0.0001
VD/VT mean ± SD	0.21 ± 0.04	0.19 ± 0.05	0.010
Peak ventilation (L/min), mean ± SD	48 ± 12.5	57.8 ± 17	<0.0001
Tidal peak volume (L), mean ± SD	1.58 ± 0.42	1.76 ± 0.51	0.001
RR peak (b/m), mean ± SD	31.1 ± 6.4	33.3 ± 6.4	0.006
Ventilatory oscillation (*n*, %)	33 (25)	11 (8)	0.0004

AT, anaerobic threshold; AT-VO_2_, anaerobic threshold at oxygen consumption peak; b/m, beats per minute; RQ, respiratory quotient; RR peak, respiratory exchange ratio calculated as VCO_2_/VO_2_; O_2_ pulse, oxygen pulse; SD, standard deviation; VD/VT, dead space to tidal volume ratio; VE/VCO_2_ slope, minute ventilation/carbon dioxide production ratio slope; VO_2_ peak, maximum oxygen consumption; ΔVO_2_/Δwork, change in oxygen uptake and change in work rate ratio.

^a^ Patients with available data during the characterization period, which was 6 months before the index date, the date of the first prescription of sacubitril/valsartan during the inclusion period. Patient follow-up was done for at least 12 months.

## 4 Discussion

REAL.IT was a real-world, multicentric study that used the EMR of HF patients who started therapy with sacubitril/valsartan in nine specialist centers in Italy. The study aimed to provide insights into the utilization of sacubitril/valsartan and its changing clinical patterns. A total of 924 patients were evaluated, with their demographic (mean age, 65 years; 85% male) and clinical characteristics similar to those of the population of other international studies reporting patients' experience with sacubitril/valsartan. For example, the mean age of the patients who participated in REAL.IT was similar to those enrolled in the pivotal PARADIGM-HF study (13) and slightly lower than those of the participants in real-world studies of sacubitril/valsartan utilization (14). The full baseline demographic, clinical characteristics, and clinical outcomes of the REAL.IT study have recently been published, reporting that the therapy improved the New York Heart Association (NYHA) class in 37.5% of the patients after 5 months (10).

In line with this improvement in the clinical characteristics, this study observed improvements in the functional capacity of patients initiating treatment with sacubitril/valsartan.

A number of cardiopulmonary exercise tests have been developed to objectively assess the physical functional capacity of patients, including those with chronic HF. These include the measures utilized in REAL.IT: peak VO_2_, which is a measure of maximal exercise capacity; and VE/VCO_2_ slope, which is a measure of ventilatory efficiency (15–19). The improvements in these parameters have been shown to bear a prognostic value in HF (15, 17, 18, 20–22).

Several studies have shown improvements in the objective exercise capacity and tolerance measures of patients with symptomatic HF after the sacubitril/valsartan initiation (23–33). Palau et al. (23) and Vitale et al. (24) showed improvements in the peak VO_2_ and VE/VCO_2_ slope in HFrEF patients after follow-up durations between 1 and 6 months after the sacubitril/valsartan initiation (i.e., the peak VO_2_ improved from 14.6 ± 3.3 to 17.2 ± 4.7 ml/kg/min; *p* < 0.0001, and the VE/VCO_2_ slope decreased from 34.1 ± 6.3 to 31.7 ± 6.1; *p* = 0.006). Similarly, a study in a smaller Italian cohort showed that sacubitril/valsartan treatment improved the cardiopulmonary response to exercise in HF patients (i.e., the peak VO_2_ increased from 15.8 ± 3.4 to 17.0 ± 4.0 ml/kg/min) (28). Cacciatore et al. (34) also demonstrated that the main effect of sacubitril/valsartan is the improvement of functional performance, including PF together with 6MWT and VO_2_ max, and a reduction in the PAPs, E/E′, VE/VCO_2_ slope, and NT-proBNP. Correspondingly, in REAL.IT, sacubitril/valsartan significantly improved the peak VO_2_, which, together with a decrease in the VE/VCO_2_ slope, indicated an overall improvement in the functional and cardiopulmonary capacities of the patients. Sacubitril/valsartan has also been shown to benefit a 6-min walk distance (25–27, 35–37).

The measures of cardiopulmonary testing, namely, the peak VO_2_, the VE/VCO_2_ slope, and the 6-min walk test, provided valuable prognostic information for HF mortality and morbidity (15, 22); therefore, we propose herein that they all be included as routine components of clinical studies designed to assess the impact of therapeutic interventions on patients with HFrEF. These measures could help enhance management options and optimize outcomes in HF patients.

This study was a retrospective observational study that is reliant on anonymized data sourced from EMR. The analyses were subject to limitations inherent in this study design, particularly relying on precise patient data registration. Although steps were taken to account for this in the analyses, some patients had incomplete available data (10, 11). For example, underestimated events could be because they were not routinely registered in the electronic records. This could also lead to a potential underreporting of the outcomes analyzed in the study. However, these data on prescription and adherence to therapy cannot be referred to the entire national territory. The patients included in the analysis were referred to highly specialized HF centers in Italy. In this setting, the prescription of sacubitril/valsartan is likely more widespread, and cardiologists could have initiated this treatment earlier than under different circumstances. Therefore, our patient population may not completely represent patients under typical primary care settings, and the applicability of our findings to the broader population may somewhat be limited (10, 11). Nevertheless, this was a multicenter study that involved several Italian centers with relevant expertise in the management of HF.

Another limitation of this work is the unavailability of a correlation analysis between CPET and other clinical functional and laboratory variables, such as LVEF, NYHA classification, and NT-proBNP levels, for the subpopulation analyzed.

Overall, however, the data along with the observed protective effects of sacubitril/valsartan in the initial year of follow-up indicate that the initiation of this treatment was an effective management approach for patients with HFrEF. This strategy demonstrated notable benefits in terms of functional and cardiopulmonary capacities.

The retrospective nature of this analysis was indeed the main limitation of this study, and the analysis of the functional capacity was conducted on only 170 patients. Another limitation is the lack of a control group. However, following the current guidelines, it would have been ethically unjustifiable to withhold treatment from patients with HFrEF. Selecting a control group with contraindications to sacubitril/valsartan therapy is also considered methodologically incorrect because the two populations under examination would have been highly heterogeneous.

Finally, a further limitation of this study is the follow-up duration of approximately 12 months. It would be desirable to observe the effects of the drug on a patient's long-term functional capacity.

## 5 Conclusions

The practical, real-world data presented in this study demonstrate that the initiation of sacubitril/valsartan in patients with HF under daily clinical settings in Italy leads to enhanced functional and cardiopulmonary capacities, which suggests effective therapeutic management.

## Data Availability

The raw data supporting the conclusions of this article will be made available by the authors, without undue reservation.

## Appendix

**Appendix 1 Principal investigators of the REAL.IT study**.

Dr. Andrea Di Lenarda, SC Cardiovascolare e Medicina dello sport Azienda Sanitaria Universitaria Giuliano Isontina (ASU GI), Trieste, Italy;

Dr. Andrea Passantino, U.O. Cardiologia ICS Maugeri Bari SpA SB IRCCS Istituto di Bari, Bari, Italy;

Dr. Alessandra Villani, U.O. Day Hospital—MAC Cardiologia Acuto/Riab Istituto Auxologico Italiano—Ospedale S. Luca, Milano, Italy;

Dr. Claudio Bilato, U.O.C. Di Cardiologia Azienda ULSS 8 Berica—Ospedali dell'Ovest Vicentino, Arzignano, Italy;

Dr. Michele Correale, SC Universitaria di Cardiologia AOU “Ospedali Riuniti” Foggia, Foggia, Italy;

Dr. Gabriele Di Gesaro, U.O. Cardiologia IRCCS ISMETT Palermo, Palermo, Italy;

Dr. Daniela Miani, SOC Cardiologia, Dipartimento Cardiotoracico Azienda Sanitaria Universitaria Friuli Centrale Ospedale S Maria della Misericordia, Udine, Italy;

Dr. Filippo Maria Sarullo, U.O.S.D. di Riabilitazione Cardiovascolare Ospedale Buccheri La Ferla Fatebenefratelli, Palermo, Italy;

Prof. Erberto Carluccio, Cardiologia e Fisiopatologia Cardiovascolare Azienda Ospedaliera Universitaria “Santa Maria della Misericordia”, Perugia, Italy.
